# *UGT2B17* Genotype and the Pharmacokinetic Serum Profile of Testosterone during Substitution Therapy with Testosterone Undecanoate. A Retrospective Experience from 207 Men with Hypogonadism

**DOI:** 10.3389/fendo.2013.00094

**Published:** 2013-07-29

**Authors:** Anne Kirstine Bang, Niels Jørgensen, Ewa Rajpert-De Meyts, Anders Juul

**Affiliations:** ^1^Department of Growth and Reproduction, Faculty of Health and Medical Sciences, Rigshospitalet, University of Copenhagen, Copenhagen, Denmark

**Keywords:** *UGT2B17*, testosterone, testosterone undecanoate, hypogonadism, testosterone treatment

## Abstract

**Background:** Testosterone (T) is mainly excreted in the urine as testosterone glucuronide (TG). This glucuronidation is partly dependent on the *UGT2B17* genotype, and TG excretion is therefore lower in men having the *UGT2B17* deletion. However, the possible influence of *UGT2B17* genotype on serum T during androgen therapy is unknown. We retrospectively investigated the possible association between the *UGT2B17* gene polymorphism and serum T levels in hypogonadal men during Testosterone undecanoate (TU) substitution therapy.

**Subjects and Methods:** Two hundred and seven patients treated with TU (Nebido^®^) were genotyped by quantitative polymerase chain reaction for the *UGT2B17* deletion polymorphism. All were given 1000 mg TU per injection at 0, 6, and 18 weeks. Blood samples were taken 2 and 6 weeks after the first and second injection, prior to the third injection, and after 2–3 years of treatment. We analyzed for the levels of T, luteinizing hormone (LH), sex-hormone-binding globulin, estradiol, prostate specific antigen, hematocrit, hemoglobin, and total cholesterol.

**Results:** The *UGT2B17* genotype frequency was: ins/ins: 42%, ins/del: 44%, and del/del: 14%. During the initial 18 weeks of TU treatment, large intra- and inter-individual variations in serum T levels were observed. Large peaks in T levels, ranging from 6.7 to 69.5 nmol/l, were noted 2 weeks after injections, regardless of the genotype. T levels did not differ between the three genotypes prior to the third injection, but the del/del group had significantly lower levels of LH. At follow-up after 2–3 years, the injection interval or daily T dosage was not dependent on the *UGT2B17* genotype.

**Conclusion:** In conclusion, we found large intra- and inter-individual variations in serum T during standard TU treatment regimen in hypogonadal men. Only subtle differences in serum T and LH were noted according to *UGT2B17* genotype, which however suggest that the *UGT2B17* genotype exert modest influence on the pharmacokinetic profile of T after TU treatment.

## Introduction

The male sexual hormone Testosterone (T) became available for clinical use approximately 70 years ago (1). Since then, the increase in the clinical use of T in treatment of men with androgen deficiency, but also its illicit use as a performance enhancer in sports has resulted in increased research activity on factors influencing the metabolism and excretion of T. A commonly used method to screen for possible illicit intake of T is based on the urinary ratio of T-glucuronide (TG) and epitestosterone glucuronide, often referred to as the T/E-ratio.

In 2006 a novel discovery was made when Jakobsson and co-workers demonstrated that a common polymorphism in the *UGT2B17* gene influenced the excretion of T in the urine (2). The *UGT2B17* gene encodes an enzyme that belongs to a large family of UDP-glucuronosyltransferases that catalyzes the transfer of glucuronosyl group from uridine 5′-diphospho-glucuronic acid to a large variety of substrates including steroid hormones [for review, see Ref. (3)]. This glucuronidation results in more polar and hydrophilic steroids, and hereby facilitates their elimination through bile and urine. The *UGT2B17* polymorphism is a copy number variation, occurring in populations with variable frequency. The homozygous deletion is much more common in the Korean (67%) and Chinese (77%) population than, e.g., the Swedish (9%) and Danish (9%) populations (2, 4, 5). Men carrying one or two alleles of the *UGT2B17* gene excrete significantly greater (8 and 13 times, respectively) amounts of TG in the urine than men homozygous for the deletion (6). Testosterone injections to healthy subjects homozygous for the *UGT2B17* deletion are not detected by the expected increased T/E-ratio in urine samples, and this has drawn great attention in the cases of anabolic steroid abuse by male athletes (6, 7).

Several studies with focus on the urinary excretion of T and serum T levels according to *UGT2B17* genotypes in normal men have been published (2, 5–7). However, to our knowledge, no studies have investigated a possible association between *UGT2B17* genotypes and serum T levels in hypogonadal men during T replacement therapy with long-acting testosterone undecanoate (TU).

Our hypothesis was that the hypogonadal patients carrying the *UGT2B17* deletion may respond differently to the testosterone treatment, and this may be reflected in changed steroid hormone profiles, especially serum testosterone, which may prompt dose adjustments. We also wanted to know whether *UGT2B17* genotype should be taken into account when starting T substitution therapy in these patients. To address this question we investigated retrospectively the *UGT2B17* genotypes in a group of hypogonadal males treated with TU, and correlated the genotypes to their comprehensive hormone profiles and individual treatment regimen, measured at several time points during therapy.

## Materials and Methods

### Patients and treatment

We retrospectively identified 376 men treated in our clinic with long-acting TU (Nebido^®^) due to hypogonadism in the period 2005–2010. All patients, except 10, had been treated with other testosterone products prior to TU and did not have a wash-out phase before starting TU treatment. The patients were all Danish citizens, predominantly Caucasian, but ethnicity was not accounted for. The median age of the 207 men included in the study was 39.2 years (range 15.2–71.6 years) at the treatment start.

**Figure 1:**
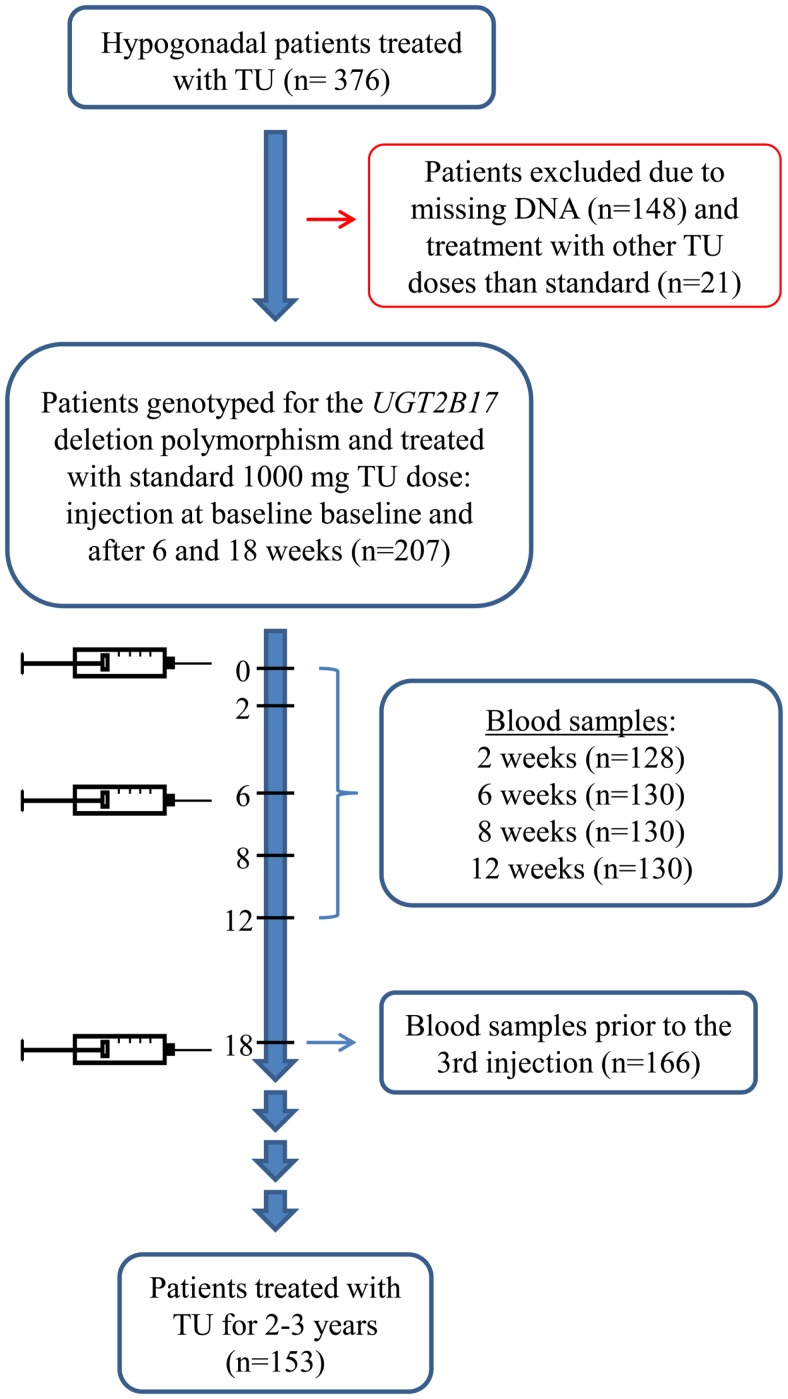
**Flowchart of the patients retrospectively included in the study**.

DNA samples were available from 228 of these men and they were genotyped for the *UGT2B17* deletion polymorphism. Two hundred and seven of these patients had initially been treated with a standard dose of 1000 mg TU in 4 ml oily solution per injection, and the second and third injections were given 6 and 18 weeks after the initial. The remaining 21 patients had been given other doses than the standard 1000 mg TU and were therefore excluded from the final analysis. Flowchart of the patients included and excluded in the study is depicted in Figure 1.

In our clinic the standard regimen for blood sample control is at baseline – at the start of TU treatment, and after 2, 6 (prior to second injection), 12, and 18 (prior to third injection) weeks. After the first three injections, the patient’s treatment was evaluated based on serum levels of total T, hemoglobin, blood pressure as well as clinical symptoms. Subsequent evaluations were done at control visits at 6–9 months later, where the patients were seen in our outpatient clinic and had blood samples drawn prior to a new injection. Changes in the individual treatment regimen were done by adjusting the interval between the injections from 10 to 18 weeks. When the patients were considered well-adapted to therapy, the subsequent injections were mainly given by the patient’s general practitioner and follow-up at the outpatient clinic was done approximately once a year.

Of the 207 patients, 130 patients had most of the blood samples drawn according to our standard regimen and could be included in the analysis of hormone fluctuation during the first 18 weeks of treatment. One hundred and twenty patients had blood samples drawn at all the standard time points, eight patients had blood samples drawn at: 0, 2, 6, 8, and 12 weeks (missing the last control), and two patients at: 2, 6, 8, 12, and 18 weeks (missing the baseline control). In the analysis on hormone levels prior to the third injection we included a total of 166 patients, 122 of the previously mentioned patients that had followed standard regimen as well as 44 patients who only had blood samples drawn approximately 18 weeks after the initial injection, prior to the third injection (Figure 1).

Of the 207 men, 153 men had been treated with TU for 2–3 years when our present analysis was performed.

The blood sampling, clinical, and biochemical analyses were performed as part of our standard clinical routine. The patient record files were evaluated retrospectively and the relevant information was extracted in unidentifiable format. The study did not require any contact with patients, and must in accordance with Act 2003-05-28 no. 402 be defined as a retrospective study, solely entailing registration of specific data regarding the patient. The study was reported to the Danish Data Protection Agency. Ref. no: 2007-41-006.

### Hormone and hematological tests in blood samples

Blood samples were drawn to assess the levels of T, luteinizing hormone (LH), estradiol, and sex-hormone-binding globulin (SHBG) as well as hemoglobin, hematocrit, Prostate Specific Antigen (PSA), and cholesterol. Serum levels of T were determined using a time-resolved fluoroimmunoassay (Delfia, Wallac, Turku, Finland). LH and SHBG were determined using a time-resolved immunofluorometric assay (Delfia, Wallac, Turku, Finland), and estradiol by radioimmunoassay (Pantex, Santa Monica, CA, USA). The intra- and inter-assay coefficient of variations (CVs) for measurement of both T and SHBG were<8 and<5%, respectively. CVs for LH were 3 and 4.5%, respectively and 7.5 and 13% for estradiol (8) free T was calculated by the Vermeulen (FT) formula (9).

Prostate Specific Antigen was determined by Sandwich Electrochemiluminescence-immunoassay (ECLIA), CV max. 7%. Cholesterol was measured enzymatically and by absorbance spectrophotometry, CV max. 5%. Hematocrit was measured by Reed Blood Cell (RBC) detector counts via Hydro Dynamic Focusing and calculated via the RBC pulse height detection method. CV max. 2.3–2.5%. Hemoglobin was measured by chemical color reaction, absorbance spectrophotometry, CV max. 1.5–2%. All tests were performed by standard procedures at the local clinical biochemistry department. These parameters were only assed prior to the third injection.

### *UGT2B17* genotyping

DNA was isolated from leukocytes from peripheral blood using a semi-automatic method on the QuickGene-810 Nucleic Acid Isolation System with the QuickGene DNA whole blood kit (Fujifilm, Life Science Products, Tokyo, Japan). The concentration and quality of DNA were assessed using a NanoDrop ND-1000 Spectrophotometer (Saveen Werner AB, Malmö, Sweden). To establish the number of copies of *UGT2B17* gene, quantitative polymerase chain reaction (qPCR) with specific *UGT2B17* primers was performed on the Mx3000P platform (Stratagene, Cedar Creek, TX, USA) according to the protocol described previously in detail (5).

The three genotypes for the *UGT2B17* gene polymorphism were noted as the wild type (ins/ins), the heterozygous deletion (ins/del), and the homozygous deletion (del/del).

## Statistics

Kruskal–Wallis non-parametric test was performed to compare the distribution of diagnoses as well as age and former testosterone treatment among the 207 patients stratified according to the three possible genotypes.

Hormone levels as well as hemoglobin, hematocrit, total cholesterol, and PSA were compared among the three *UGT2B17* polymorphism groups (ins/ins, ins/del, del/del) by linear regression models (ANOVA). Hormone and PSA levels were transformed by the natural logarithm to obtain normal distribution of the residuals. This transformation resulted in a better approximation of a normal Gaussian distribution. Hematocrit, hemoglobin, and total cholesterol were all normally distributed. Patients with hypogonadotropic hypogonadism were excluded from any analyses of the LH levels.

The associations between the *UGT2B17* genotype and the different hormone levels as well as hemoglobin, hematocrit, PSA, and total cholesterol levels were investigated at two different time points. The first time-point constituted of the approximately first 18 weeks of TU treatment. We investigated the fluctuation of T and estradiol during the 18 weeks, ΔT levels calculated as the difference from week 8 to 18 after initial treatment and blood samples prior to the third injection. The second time-point was after approximately 2–3 years of individualized TU treatment. In the regression analysis we accounted for age, previous testosterone treatment as well as the basic diagnosis as possible confounders. Age was statistically associated with the levels of free T, SHBG, and cholesterol and therefore included in the final analyses. Previous testosterone treatment was found to be statistically associated with LH and therefore was also incorporated in the statistical model.

After 2–3 years of individualized TU treatment (approximately after 9–11 injections), we compared the individual injection intervals as well as the daily T dosage between men in the three *UGT2B17* genotype groups. The daily T dosage 2–3 years after initial TU injection was calculated as the [dosage (mg)/the interval (days)]. Difference between injection interval as well as daily T doses in the three groups was tested by linear regression analysis.

SPSS version 19.0 for Windows was used to perform all statistical analysis.

## Results

### Distribution of *UGT2B17* genotypes

Twenty-nine of the 207 patients (14%) had a homozygous deletion (del/del), 91 (44%) were heterozygotes (del/ins), and 87 (42%) were wildtype for the *UGT2B17* gene (ins/ins). These genotype frequencies were in accordance to Hardy–Weinberg equilibrium and similar to those reported previously in Caucasian populations.

There was no significant difference in age between men in the three genotype groups.

Table 1 shows the distribution of diagnoses stratified according to genotype. The distribution of diagnostic groups (*p* = 0.6) or age (*p* = 0.84) did not differ between men from the three *UGT2B17* groups.

**Table 1:** **Primary diagnoses stratified according to the *UGT2B17* genotype**.

Diagnoses	ins/ins *n* = 87 (42.0%)	ins/del *n* = 91 (44.0%)	del/del *n* = 29 (14.0%)	Total *n* = 207 (100%)
Klinefelter syndrome (KS) and 46XX male	16 (39.0)	20 (48.8)	5 (12.2)	41 (100)
Primary testicular disease, excl (KS)	46 (42.6)	49 (45.4)	13 (12.0)	108 (100)
Kallmann syndrome	10 (45.5)	6 (27.3)	6 (27.3)	22 (100)
Hypo. hypogonadism (other reasons)	4 (40.0)	5 (50.0)	1 (10.0)	10 (100)
Irradiation-induced hypogonadism	3 (50.0)	3 (50.0)	0	6 (100)
Others*	8 (40.0)	8 (40.0)	4 (20.0)	20 (100)

*Injury/torsion/aplasia of the testis (n = 7), Fragile X syndrome (n = 1), Charge syndrome (n = 1), Kennedy syndrome (n = 1), Partial androgen insensitivity syndrome (n = 3), Y-microdeletion (n = 1), Liver transplant (n = 1), Heart transplant (n = 1), HIV (n = 3), and hypogonadism induced by opiods (n = 1).

### Response to TU during the first 1–18th weeks of treatment (first–third injection)

**Figure 2:**
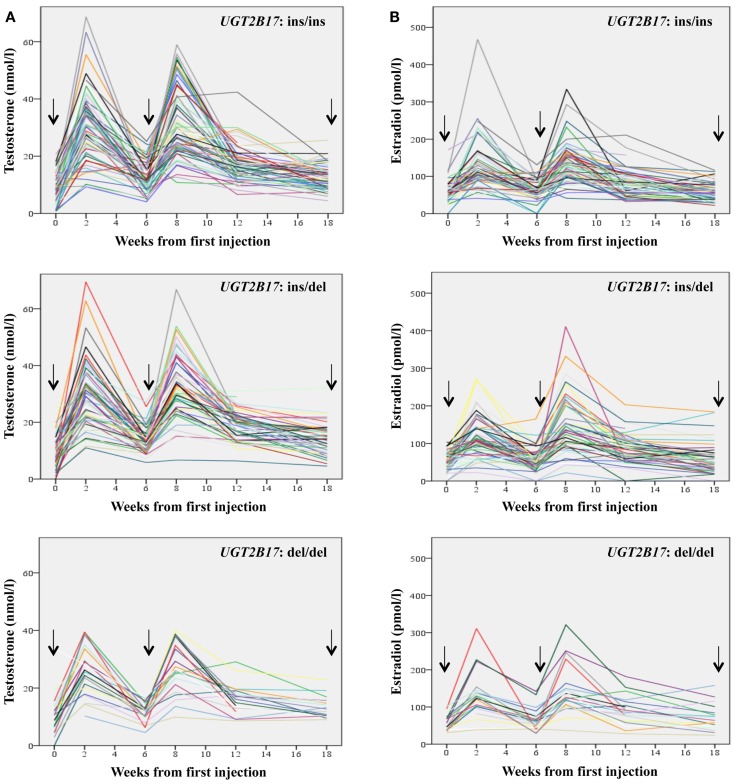
**Fluctuation of the levels of Testosterone (A) and Estradiol (B) during the first 18 weeks of treatment with Testosterone Undecanoate stratified according to the *UGT2B17* genotype**. Arrows indicate the three injections.

Individual serum levels of T and estradiol before and during the three TU injections according to *UGT2B17* genotype are depicted in Figures 2A,B, respectively. A large inter-individual variation in both hormone levels was noted at the blood sampling time during the entire 18 week period. At the blood sampling times approximately 2 weeks after the first and second injection, the serum levels of T were high, with a total range from 9.3 to 69.5 nmol/l during the first peak and from 6.7 to 66.8 nmol/l during the second peak. There were no significant differences in serum T levels at these peaks between the three *UGT2B17* genotype groups (*p* > 0.05).

The increase in T values from week 8 to 18 (ΔT) after initial treatment differed significantly between the three genotype groups; men belonging to the *UGT2B17* del/del group had a smaller ΔT values (median: 10.9 nmol/l) than the ins/del (median: 14.3 nmol/l) and ins/ins group (median: 18.3 nmol/l) (*p* = 0.001 and *p* = 0.003, respectively).

In accordance with T levels, estradiol levels also showed large inter-individual variations as well as high levels at the blood sampling times approximately 2 weeks after the first and second injection. The serum levels of estradiol were high, with a total range from 23 to 468 pmol/l during the first peak and from 22 to 411 pmol/l during the second peak. There were no significant differences in serum estradiol levels at these peaks between the three *UGT2B17* genotype groups *p* > 0.05.

A total of 166 patients had blood samples drawn prior to the third injection, approximately after 18 weeks of treatment. Hormone levels, hemoglobin, hematocrit, total cholesterol, and PSA levels stratified according to *UGT2B17* genotypes as well as statistical results are shown in Table 2.

**Table 2:** **Biochemical parameters in hypogonadal men receiving Testosterone Undecanoate (TU) injections**.

	1. ins/ins		2. ins/del		3. del/del		*p*-Values			
	Mean (SD)	Median (5−95% percentile)	Mean (SD)	Median (5−95% percentile)	Mean (SD)	Median (5−95% percentile)	Total	1 vs. 2	1 vs. 3	2 vs. 3
**PRIOR TO THE THIRD INJECTION**										
Testosterone (nmol/l)	14.2 (5.6)	13.8 (6.6−23.8)	12.8 (4.3)	12.8 (6.7−20.8)	14.1 (4.5)	13.2 (9.0−26.1)	0.23	0.13	0.80	0.21
Free testosterone (pmol/l)	286 (112)	275 (136−498)	275 (90)	268 (130−463)	323 (95)	316 (183−497)	0.24	0.66	0.17	0.09
SHBG (nmol/l)	35 (19)	31 (14−76)	32 (21)	28 (11−59)	27 (12)	26 (10−52)	0.20	0.14	0.14	0.60
LH (U/l)*	7.6 (10.7)	2.4 (0−30.6)	8.22 (11.8)	3.6 (0−39.0)	2.7 (5.1)	0.5 (#1)	**0.02**	0.43	**0.005**	**0.02**
Estradiol (pmol/l)	61 (38)	61 (19−148)	59 (26)	54 (26−114)	76 (46)	61 (24−201)	0.29	0.96	0.14	0.13
Estradiol/testosterone	4.6 (3.1)	3.8 (1.3−10.0)	4.7 (1.6)	4.3 (2.2−7.8)	5.4 (2.7)	4.7 (2.4−11.9)	0.25	0.25	0.13	0.43
Hematocrit	0.45 (0.04)	0.45 (0.38−0.5)	0.44 (0.03)	0.44 (0.39−0.5)	0.46 (0.04)	0.46 (0.55−0.4)	0.21	0.75	0.13	0.08
Hemoglobin (mmol/l)	9.5 (0.8)	9.6 (8.0−10.6)	9.5 (0.6)	9.5 (8.6−10.7)	9.9 (0.9)	10.0 (#2)	0.23	0.90	0.12	0.10
Total cholesterol (mmol/l)	4.8 (1.1)	4.8 (3.6−6.6)	5.0 (1.2)	5.0 (3.0−7.3)	4.8 (1.1)	4.4 (#3)	0.61	0.33	0.61	0.92
Prostate specific antigen (μg/l)	0.7 (0.38)	0.6 (0.2−1.5)	0.8 (0.63)	0.7 (0.2−1.97)	0.94 (1.4)	0.5 (#4)	0.73	0.43	0.73	0.85
**AFTER 2−3 YEARS OF TREATMENT**										
Daily dosage of TU mg/day	11.73 (1.1)	11.9 (9.2−14.1)	11.99 (0.9)	11.90 (10.2−14.3)	11.83 (0.9)	11.90 (9.7−14.2)	0.34	0.14	0.69	0.54
Injection interval (weeks)	12.23 (1.2)	12.0 (10.2−13.0)	11.99 (0.9)	12.0 (10.0−14.0)	12.14 (0.9)	12.0 (10.10−14.8)	0.47	0.22	0.74	0.62

*Data are presented prior to the third injection as well as calculated daily testosterone dosage and injection interval after 2–3 years of individualized treatment. All data is stratified according to the UGT2B17 genotype. Linear regression models (ANOVA) were used to compare the different levels between the three genotypes groups (p-values). Bold indicating p-value < 0.05*.

*LH levels from patients with Hypogonadotropic hypogonadism were not included.

*#Range from 1: 0–15.4 (n = 13), 2: 8.2–11.8 (n = 16), 3: 3.3–6.8 (n = 13), 4: 0.2–0.6 (n = 17)*.

No significant difference was detected in T levels between the three groups (*p* = 0.23), neither in a simple analysis, nor in pair-wise comparison between the individual groups. The levels of the calculated free T showed a non-significant (*p* = 0.24) tendency toward lower levels according to the number of *UGT2B17* alleles. LH levels differed significantly between the three genotypes. Patients homozygous for the deletion had significantly lower levels of LH prior to the third injection compared to the heterozygous and wildtype men (median; del/del: 0.5 U/l, ins/del: 3.6 U/l, and ins/ins: 7.6 U/l), *p* = 0.02 and *p* = 0.005, respectively. SHBG, hematocrit, hemoglobin, cholesterol as well as PSA levels did not differ between *UGT2B17* genotype.

### Long term (2–3 years) response to TU treatment

Of the 207 men, 153 men had been treated with TU for 2–3 years when our present analysis was performed, with the following *UGT2B17* genotype distribution: ins/ins: *n* = 62 (40.5%), ins/del: *n* = 70 (45.8%), del/del: *n* = 21 (13.7%). One patient in the ins/ins group had his TU dosage reduced to 750 mg, the rest of the patients were still given 1000 mg but with different injection interval if needed. At this time-point the individual injection intervals ranged from 10 to 18 weeks and the calculated daily TU doses from 7.94 to 14.29 mg for all 153 men. The majority of the patients had injection intervals of 12 weeks (55.6%) or larger than 12 weeks (13 weeks: 20.9%, 14 weeks: 2.6%, 15 weeks: 0.7%, 16 weeks: 0.7%, and 18 weeks: 0.7%). Approximately 18.9% had injection intervals shorter than 12 weeks with the distribution of 11 weeks: 13.7% and 10 weeks: 5.2%. The mean and median injection intervals as well as daily testosterone dosages stratified according to *UGT2B17* genotype are shown in Table 2. No differences in the injection interval or daily dosage between the three genotypes were detected.

We did not find a significant association between the later interval regime and the ΔT values during the first 18 weeks of treatment.

## Discussion

In this large study of hypogonadal men, we addressed a hypothesis that carriers of the *UGT2B17* deletion polymorphism may obtain higher serum T levels during therapy with long-acting TU, and might need a lower T dosage than men with both alleles present. We did not corroborate our hypothesis with regard to the nadir levels of serum T prior to the third injection. *UGT2B17* genotype influenced serum T and LH levels only marginally. Interestingly, we did find that men who carried a homozygous *UGT2B17* deletion had lower LH levels prior to the third injection and a smaller decline in T levels from the peak levels obtained after the second injection to the nadir levels prior to the third injection. This could imply a slower T excretion. We speculate that despite that the serum T levels were not significantly increased, subtle changes in the negative feedback might have been sufficient at the hypothalamic-pituitary level to decrease the release of LH. However, the *UGT2B17* genotype did not influence the response to individualized TU treatment regimen, following the initial 18-weeks fixed treatment regimen, when the daily dosage and TU injections were adjusted.

A few previous studies have investigated the association between *UGT2B17* polymorphism and reproductive hormone levels in serum and found no association between the baseline levels of T in serum, and *UGT2B17* genotype (2, 5, 10). In our own study of Danish pubertal boys, no increase in T level or decrease in LH were found, despite that a deletion of the *UGT2B17* gene was associated with a significantly lower level of TG in the urine (5). One study found that Chinese young men with the del/del genotype had significantly higher levels of both T and estradiol compared to men carrying one or two alleles (4). To our knowledge only a few studies have investigated the association of serum levels of T after exogenous T administration (11, 12). Ekström and co-workers found no association between *UGT2B17* genotypes and serum levels of T both baseline and 2, 4, and 15 days after a single intramuscular exogenous T enanthate injection (11, 12).

If serum levels of T are not elevated in individuals lacking the *UGT2B17* gene – what is then the metabolic fate of T? It could be speculated that men carrying the deletion polymorphism may have a larger conversion of T to either dihydrotestosterone (DHT) or estradiol. However in two studies (6, 12) no significant differences in serum levels of DHT or urinary concentrations of glucuronides of major final metabolites of DHT were found. Estradiol is rarely described in studies concerning the *UGT2B17* gene, but the previously mentioned Chinese study found higher levels of estradiol along with T in the del/del group (4). This was not the case in the patients examined in our current study or our former study in pubertal boys (5).

The metabolism of TU is likely to be modulated by other factors, in addition to *UGT2B17*. TU is a T ester that has to be hydrolyzed to the active T, and factors that alter the hydrolyzation could also influence serum T levels. It is still unknown which enzymes catalyze this reaction. The phosphodiesterase 7B (PDE7B) has earlier been linked to the hydrolysis of testosterone enanthate, and polymorphisms in this *PDE7B* gene can influence the levels of serum T (11). Whether this polymorphism or variants in other enzymes involved in the hydrolysis could also affect serum T levels after TU administration remains unknown and requires further studies.

A minor part of T is excreted as sulfate conjugates (TS), and it is plausible that in the absence of the *UGT2B17* glucuronidation, the sulfation would be up-regulated. However previous studies have shown that 95% of testosterone sulfate (TS) is of testicular origin and did not find significantly higher levels of TS in accordance to lower levels of TG (7, 13).

Another important factor that should be taken into account is a possibility that diet or drugs may alter or inhibit the glucuronidation of T. Recent studies have shown that catechins from tea extract, phenolic compounds in red wine as well as two non-steroid anti-inflammatory drugs (NSAIDs), diclofenac and ibuprofen, can inhibit the glucoronidation of T *in vitro* (14–16). Although NSAIDs have not shown the same effect *in vivo*, it remains to be investigated whether there are other possible common dietary substances that could have an influence on the glucuronidation of T (17).

In this study we also analyzed T levels during the first 18 weeks of TU treatment in a large group of hypogonadal men and noted a wide inter- and intra-individual variation during the entire period. TU was initially licensed in 2005 and since then several follow-up studies of the pharmacokinetics and effects of this treatment have been completed (18). The generally accepted treatment regimen starts with an initial dose of 1000 mg TU followed by a second injection 6 weeks later and hereafter every 12 weeks (19). In this study we observed that men in the *UGT2B17* del/del group experienced smaller declines in their serum T after injections. To our knowledge this is the first time the inter-individual variation of T throughout the initial 18 weeks of TU treatment was investigated in correlation to *UGT2B17* genotype. *UGT2B17* genotype influenced serum T and LH levels marginally in our hypogonadal patients.

A former study by Moisey et al. has recommended that clinicians use age and body size when estimating dosing frequency in treatment with TU (20) and the study by Ekström and co-workers also found that serum levels of T after exogenous T administration were associated to body weight (11). We found a significant association between age and free T but unfortunately there were too few available weight measurements, thus we were not able to include BMI as a confounder in any of the analysis, although this would have been relevant.

In conclusion, we found large intra- and inter-individual variations in serum T during standard TU treatment regimen in hypogonadal men, but noted only subtle differences in serum T and LH in different *UGT2B17* genotypes. These subtle changes are likely due to lower T excretion rates in homozygous carriers of the deletion, and suggest that *UGT2B17* may influence the pharmacokinetic profile of T. These observations need to be further investigated, before *UGT2B17* genotyping can be recommended as a clinically relevant test in relation to TU treatment.

## Conflict of Interest Statement

The authors declare that the research was conducted in the absence of any commercial or financial relationships that could be construed as a potential conflict of interest.
